# The Spondyloarthritides: From Scientific and Therapeutic Immobility to the Forefront of Rheumatology Research

**DOI:** 10.31138/mjr.121224.sat

**Published:** 2024-12-31

**Authors:** George E. Fragoulis, Charalampos Papagoras

**Affiliations:** 1 Joint Academic Rheumatology Program, First Department of Propaedeutic and Internal Medicine, National and Kapodistrian University of Athens, Athens, Greece;; 2 First Department of Internal Medicine, University Hospital of Alexandroupolis, Democritus University of Thrace, Alexandroupolis, Greece

**Keywords:** spondyloarthritis, axial spondyloarthritis, ankylosing spondylitis, psoriatic arthritis

The recent history of spondyloarthritides (SpA), particularly axial spondyloarthritis (AxSpA) and psoriatic arthritis (PsA), is that of scientific commitment and determination to understand the disease and offer relief to patients. Indeed, it was hardly 30 years ago that medical textbooks included no other treatment for ankylosing spondylitis than nonsteroidal anti-inflammatory drugs and physiotherapy with the wishful prospect that gradual ankylosis would allow an acceptable functional state.

Psoriatic arthritis became widely recognised as a distinct disease following the seminal paper by Moll and Wright in 1973.^1^ However, for the next 40 years, it continued to be treated as a poor relation of rheumatoid arthritis (RA), since all scientific research and clinical management relied on experience with RA: conventional synthetic disease-modifying anti-rheumatic drugs (csDMARDs) and anti-TNFŞ agents were introduced in PsA following their initial success in RA; measures of RA disease activity and response, like ACR 20/50/70 or DAS28, were used for PsA clinical trials, despite that PsA is commonly an oligoarthritis affecting lower limbs, which are ignored in DAS28.^2–4^ Other aspects of the disease, like axial involvement, were only rarely addressed.^5^

Nowadays, the landscape in SpA has profoundly changed. The concept of axSpA has expanded to include what is essentially an inflammatory disease of the axial skeleton, whether it is radiographically evident or not. Even the name “ankylosing spondylitis” has been changed to radiographic AxSpA, removing the ominous “ankylosis” from the patients’ outlook for the future.^6^ There are currently 3 classes of targeted treatments in AxSpA, which have offered great relief to patients and potentially delay structural progression.^7^ Concerning PsA, there are 12 classes of cs- or biologic DMARDs available (there are only 5 in RA), which offer significant musculoskeletal and profound skin responses.^8^ Finally, since both diseases adversely affect patients’ general health, not only due to their extra-musculoskeletal manifestations (e.g. uveitis, inflammatory bowel disease), but also due to their association with metabolic/cardiovascular and mental disease,^9^ clinicians are expected to manage the patients in a holistic and multi-disciplinary manner. Indeed, this approach appears to produce long-term benefits.^10^

In translational research, much progress has been made in the understanding of disease mechanisms. Non-specific stimuli, such as mechanical forces and gut dysbiosis have come into the limelight and the mechanisms linking them to aberrant immune responses are being unraveled.^11–12^ Although IL-17A has long been recognised as the main driving cytokine, the classical IL-23/IL-17A sequence has been questioned, particularly following the poor results of IL-23 inhibitors in AxSpA.^13^ Indeed, it appears that the aforementioned ubiquitous environmental insults activate locally the innate immune system including Ş/Ş T cells, innate lymphoid cells, and mucosa-associated invariant T cells to produce IL-17A, often independently of IL-23.^14–15^ These cells further recruit myeloid cells, particularly neutrophils, which mediate inflammation, but are also actively involved in a self-perpetuating inflammatory loop.^16–17^ A breakthrough was the recognition of the role of another member of the IL-17 family, namely IL-17F. In fact, innate cells are main sources of both IL-17A and IL-17F which act on the same receptors with additive inflammatory effects.^18^ Therefore, it appears that there are two pathways leading to IL-17 activity, an adoptive and an innate one, the latter being less dependent on IL-23 and more IL-17F-mediated. Consequently, the dual IL-17A and IL-17F blockade with the monoclonal antibody bimekizumab has shown high levels of efficacy across the disease spectrum (axial and peripheral musculoskeletal manifestations, skin psoriasis), verifying the pathophysiological relevance of both IL-17A and IL-17F.^19–21^

On the other hand, several issues remain to be addressed. Although the TICOSPA study failed to prove its primary hypothesis, a tight control strategy was more efficacious across several other clinical outcomes than usual care.^22^ As the treat-to-target approach is still upheld, should we reconsider the outcome measures we use in AxSpA? Further to that, the definitions of remission and low disease activity in PsA remain to be set and validated.^23–24^ On the other hand, there is a lot of discussion on how to identify and treat patients who fail multiple treatments, the so-called difficult-to-treat (D2T) patients. However, as formal definitions of D2T AxSpA and PsA are still pending, several groups have proposed their own definitions and analysed the respective populations,^25^ as in the paper by Zemrani et al. in this issue.

In translational research, new questions arise, particularly following the successful introduction of JAK inhibitors in the treatment of AxSpA and PsA. Although neither TNFα nor IL-17A/F signal through this pathway, JAK inhibitors produce quick responses comparable to those of TNFα blockers.^26^ Considering that these small molecules act upon a wide range of immune and non-immune cells and at various levels in the cytokine network, understanding how they work in SpA will help pinpoint the crucial hubs in its pathogenesis that could be specifically targeted by future drugs. Finally, the mechanisms governing both osteoanabolic processes, leading to syndesmophyte formation, and osteocatabolic ones, leading to osteoporosis, are still to be elucidated.^27^ Although pain is the everyday ailment of patients with active disease, structural damage is the long-lasting consequence permanently limiting function and quality of life.^28^ Therefore, treatments intercepting disease progression at a faster rate than those currently available will fulfil a still unmet need.^29^

To conclude, over the last 15 years, AxSpA and PsA have taken a life of their own from a research and clinical perspective. Plenty of research groups worldwide have focused their interest on those diseases, the industry supports research and the development of new drugs, while multinational scientific societies have been formed, such as ASAS, SPARTAN, and GRAPPA, to provide a field for scientific collaboration, appraisal of the literature and scientific guidance to clinicians, all aiming at the benefit of patients. The future is eagerly awaited.
